# Left temporal alpha-band activity reflects single word intelligibility

**DOI:** 10.3389/fnsys.2013.00121

**Published:** 2013-12-27

**Authors:** Robert Becker, Maria Pefkou, Christoph M. Michel, Alexis G. Hervais-Adelman

**Affiliations:** ^1^Functional Brain Mapping Lab, Department of Fundamental Neuroscience, University of GenevaGeneva, Switzerland; ^2^Brain and Language Lab, Department of Clinical Neuroscience, University of GenevaGeneva, Switzerland

**Keywords:** speech intelligibility, degraded speech, noise-vocoding, alpha oscillations, left inferior temporal cortex

## Abstract

The electroencephalographic (EEG) correlates of degraded speech perception have been explored in a number of recent studies. However, such investigations have often been inconclusive as to whether observed differences in brain responses between conditions result from different acoustic properties of more or less intelligible stimuli or whether they relate to cognitive processes implicated in comprehending challenging stimuli. In this study we used noise vocoding to spectrally degrade monosyllabic words in order to manipulate their intelligibility. We used spectral rotation to generate incomprehensible control conditions matched in terms of spectral detail. We recorded EEG from 14 volunteers who listened to a series of noise vocoded (NV) and noise-vocoded spectrally-rotated (rNV) words, while they carried out a detection task. We specifically sought components of the EEG response that showed an interaction between spectral rotation and spectral degradation. This reflects those aspects of the brain electrical response that are related to the intelligibility of acoustically degraded monosyllabic words, while controlling for spectral detail. An interaction between spectral complexity and rotation was apparent in both evoked and induced activity. Analyses of event-related potentials showed an interaction effect for a P300-like component at several centro-parietal electrodes. Time-frequency analysis of the EEG signal in the alpha-band revealed a monotonic increase in event-related desynchronization (ERD) for the NV but not the rNV stimuli in the alpha band at a left temporo-central electrode cluster from 420–560 ms reflecting a direct relationship between the strength of alpha-band ERD and intelligibility. By matching NV words with their incomprehensible rNV homologues, we reveal the spatiotemporal pattern of evoked and induced processes involved in degraded speech perception, largely uncontaminated by purely acoustic effects.

## Introduction

Despite the great acoustic variability of the speech signal, normally-hearing listeners are able to understand speech seemingly effortlessly in day-to-day situations. In order for this to be possible, the human speech perception system must be robust in the face of both natural variability and acoustic degradations. Natural variability can arise from the different realization of speech sounds by different speakers, who can be understood even though they vary in terms of their size and sex (Peterson and Barney, 1952; Smith et al., 2005), accent (Clarke and Garrett, 2004; Clopper and Pisoni, 2004; Evans and Iverson, 2004), and speech rate (Miller and Liberman, 1979), or may suffer from articulation disorders such as dysarthria (Darley et al., 1969; Kent et al., 1989). Beyond signal variations related to the speaker, external factors also have an impact on the quality of speech that is heard, such as transmission through telecommunications systems, in which case it is heavily filtered, the presence of background noise masking part of the speech signal, the presence of echoes, or the presence of multiple talkers simultaneously.

In order to investigate the mechanisms of speech perception, researchers are increasingly turning to paradigms employing artificial acoustic degradations of speech, which allow fine-grained manipulation of the level of intelligibility of a speech signal, rendering the behavioral or neural correlates of speech intelligibility amenable to investigation. Several types of manipulations have been employed, such as interrupted speech (Heinrich et al., 2008; Shahin et al., 2009), masking with noise (Davis and Johnsrude, 2003; Golestani et al., 2013), time-compression (Altmann and Young, 1993; Mehler et al., 1993; Dupoux and Green, 1997; Pallier et al., 1998; Sebastian-Galles et al., 2000), or noise-vocoding (Shannon et al., 1995). We chose to employ the latter of these, as it has been the subject of intensive research both behaviorally (e.g., Davis et al., 2005; Stacey and Summerfield, 2007; Hervais-Adelman et al., 2008, 2011; Dahan and Mead, 2010) and increasingly in neuroimaging experiments (e.g., Scott et al., 2000; Davis and Johnsrude, 2003; Scott et al., 2006; McGettigan et al., 2011; Obleser and Kotz, 2011; Hervais-Adelman et al., 2012; Evans et al., 2013).

An increasing amount of evidence regarding the neural correlates of degraded speech perception is becoming available. Functional magnetic resonance imaging (fMRI) has been extensively used to detect brain regions active while listening to degraded speech (e.g., Scott et al., 2000; Davis and Johnsrude, 2003; Giraud et al., 2004; Davis and Johnsrude, 2007; McGettigan et al., 2011; Hervais-Adelman et al., 2012; Wild et al., 2012a), while electro-encephalography (EEG) and magneto-encephalography (MEG) have been employed to study the timing of degraded speech processing through measures such as evoked response potentials and synchronized brain oscillations (e.g., Obleser and Kotz, 2011; Sohoglu et al., 2012; Peelle et al., 2013). Such experiments also provide insights into the cerebral processes that support speech perception in hearing-impaired listeners.

Neuronal oscillations are an important tool in the study of different brain functions, such as speech perception, as they address the issue of synchronization of neuronal networks within a given brain region as well as between regions, based on data obtained non-invasively and at the temporal scale neuronal activity occurs. Recent studies (reviewed by Weisz and Obleser, 2014) have focused on brain oscillations in auditory processing and speech perception. Among the different “brain rhythms”, which differ from each other on the basis of their characteristic frequency (e.g., delta oscillations are of low frequency, 1–3 Hz, while gamma oscillations have a high frequency of 30–80 Hz) and which seem to play a role in speech processing, a major stream of research has focused on the alpha rhythm (the range of 8–13 Hz). Traditionally, alpha rhythm has been considered as being highly related to visual spatial attention (Thut et al., 2006), beginning with the reports of Hans Berger in the late 1920s (Berger, 1929). Berger (1929) observed that opening the eyes lead to clear attenuation of previously visible oscillations in the 10 Hz range over occipital electrodes. Since then, numerous studies have reported findings about the close relationship of alpha rhythm activity and the visual system (e.g., Makeig et al., 2002; Becker et al., 2008; Busch et al., 2009; Mathewson et al., 2009) as well as working memory tasks (Jokisch and Jensen, 2007) to attentional and other cognitive paradigms (for a review, see Klimesch, 1999).

Despite this strong interest in alpha rhythm, much less is known about the relationship of alpha activity and auditory processing. This may be in part owed to the fact that resting alpha is much more prominent in occipital electrodes, likely detecting visual-cortical activity rather than in temporal electrodes. This might be related to the larger volume of the visual cortex compared to the auditory cortex and to the fact that it is impossible to completely deprive a healthy, hearing individual from auditory input but easy to close one’s eyes and thereby block visual input (for a review on auditory alpha see Weisz et al., 2011). The first reports of auditory-cortical oscillations in the alpha-band originate from MEG studies, in which the rhythm was initially named “tau” (Tiihonen et al., 1991; Lehtela et al., 1997). Lehtela et al. (1997) demonstrated that the presentation of short (500 ms) noise bursts was followed by decreases in alpha power, localized in the superior temporal lobes, suggesting a role for alpha as a marker of auditory processing similar to that shown in the visual system. Regarding alpha oscillations and speech perception, Krause et al. (1997) reported stronger alpha-band suppression for listening to a comprehensible text passage as when compared to listening to the same passage being played backwards (Krause et al., 1997). Despite their scarcity, such studies point to a functional role of alpha oscillations in processing speech.

In a more recent study, Obleser and Weisz (2012) investigated the role of alpha suppression in speech comprehension using degraded speech. They employed noise-vocoding, a form of artificial degradation which reduces the spectral information in the auditory signal (Shannon et al., 1995), in a graded manner, permitting the generation of stimuli on a continuum from unintelligible to intelligible, by increasing the spectral fidelity of the vocoding. The method is described in Section Stimuli below. Obleser and Weisz (2012) used words at different levels of intelligibility and hypothesized that alpha suppression should be enhanced with intelligibility, reflecting less need for functional inhibition and less effortful speech processing. This idea is compatible with the conception of alpha oscillations as a mechanism of gating or suppressing task-irrelevant high frequency oscillations (Osipova et al., 2008; Jensen and Mazaheri, 2010). In other words, a decrease in alpha power in a task-relevant region would accompany the disinhibition of gamma oscillations in the same region, which are thought to reflect active processing. In general, alpha suppression is accompanied by an increase of oscillations in the gamma band, which reflects engagement/processing as it has been shown by intracranial studies in animals and humans (e.g., Paller et al., 1987; Fries, 2005; Kaiser and Lutzenberger, 2005; Crone et al., 2006; Womelsdorf et al., 2007).

Obleser and Weisz (2012) showed a linear increase in alpha suppression as a function of word intelligibility. They interpret this finding as reflecting the listeners’ effort or ability to comprehend degraded speech. The alpha suppression was localized in superior parietal, prefrontal and anterior temporal areas. This may indicate that these brain regions are more active during the task, assuming that a local decrease in alpha power is a marker of activation of the region in question. This idea is backed by early findings of changes in resting-state alpha power as well as by numerous more recent EEG-fMRI studies which reported an inverse relationship between alpha-power and BOLD signal, under rest as well as during stimulation (Laufs et al., 2003; Moosmann et al., 2003; Goncalves et al., 2006; de Munck et al., 2007; Becker et al., 2011). Furthermore, a more recent study showed an even more direct inverse relationship between alpha-power and neuronal firing rate in monkeys (Haegens et al., 2011). The localization of the alpha-suppresion effect reported by Obleser and Weisz (2012) is partially in agreement with previous fMRI studies seeking to identify the brain regions implicated in the processing of degraded but potentially comprehensible speech (Scott et al., 2000; Narain et al., 2003; Giraud et al., 2004; Eisner et al., 2010; Wild et al., 2012a,b), revealing recruitment of brain areas involved in speech processing in general, such as bilateral superior temporal gyrus (STG) and superior temporal sulcus (STS), together with a more left-lateralized inferior-frontal and motor network.

In the present study, our aims were twofold: (1) we aimed to dissociate the EEG signatures of intelligibility and spectral complexity while listening to degraded speech, using a paradigm similar to the one published by Obleser and Weisz (2012). To this end, we asked participants to listen carefully to noise-vocoded (NV) words with different levels of degradation (therefore different levels of spectral complexity), and importantly, to the spectrally rotated version of the same stimuli. Spectrally rotated speech (Blesser, 1972) retains the same amount of spectral complexity as its non-rotated equivalent but it is incomprehensible to the naïve listener [although listeners may adapt to it after extensive training (Green et al., 2013)]. In this way, we had a spectrally-matched control condition for each level of intelligibility in order to rule out the possibility that alpha suppression is purely driven by low-level acoustic properties of the signal. Importantly, we used monosyllabic words, which are very challenging for naïve listeners to understand when degraded, in order to increase task difficulty and limit the use of top-down processes. As lexical and prosodic information facilitate comprehension and perceptual learning of degraded speech (e.g., Davis et al., 2005), by using monosyllabic words our listeners would have to rely almost exclusively on the acoustic information they could extract from the degraded input. The nature of this acoustic input was therefore essential for comprehension, given the lack of other sources of information in the stimuli and (2) by using the approach described above, we wanted to examine both induced and evoked correlates for degraded speech processing, their timing, their topography as well as the putative cerebral sources underlying the observed effects. Although the work presented here employs artificial acoustic degradation to examine the neural underpinnings of degraded speech perception, we hope that the findings will also be relevant to the cerebral processes that help to compensate for degraded auditory input in hearing impaired listeners.

## Materials and methods

### Participants

Fourteen right-handed native French speakers (two male) participated in the study. Participants self-reported as being right-handed and having no history of hearing or visual impairment. They all gave written informed consent to participate in the study, which was approved by the local ethics committee of the University Hospital of Geneva, and received financial compensation of 40 CHF.

### Stimuli

Stimuli were 360 monosyllabic French nouns, among which 36 animal names, selected from the “Lexique 3” database for French words.^1^ The animal names were used as targets in the detection task we employed. All words, apart from the animal names, were divided into three groups, matched for spoken wordform frequency (*F*_2,321_ = 0.205, *p* = 0.82; Group 1 mean = 31.94/million, Group 2 mean = 27.43/million and Group 3 mean = 28.37/million). The animal names used had mean frequency = 15.41/million. Stimulus matching was achieved using the Match program.^2^

The words were recorded in a soundproof chamber by a male native French speaker and digitized at a 44.1 kHz sampling rate. Recordings were denoised using the algorithm implemented in Audacity software^3^ and trimmed at zero-crossings preceding and succeeding the words. Root mean square (RMS) amplitude of each of the extracted stimuli was set to a fixed level, and the stimuli were then bandpass filtered between 50 and 5000 Hz using a sixth order butterworth filter. Stimuli were then NV using 1, 4, 8 and 16 frequency bands following the procedure described by Shannon et al. (1995). Stimuli were filtered into quasi-logarithmically spaced frequency bands intended to mimic the tonotopic organization of the cochlea (Greenwood, 1990) between 50 and 5000 Hz, using second order butterworth filters and full-wave rectified, producing the amplitude envelope of each band. Each envelope was convolved with noise band-pass filtered into the same frequency range as the source band. The amplitude-modulated carriers were then recombined to produce NV words. The number of frequency bands has been shown to be a crucial factor for the intelligibility of vocoded words, with higher numbers of bands producing more intelligible stimuli (Shannon et al., 1995). The four NV versions of each word were created, producing a continuum from completely unintelligible (1-band noise-vocoded, NV1) to relatively easily comprehensible (16-band noise-vocoded, NV16).

**Figure 1:**
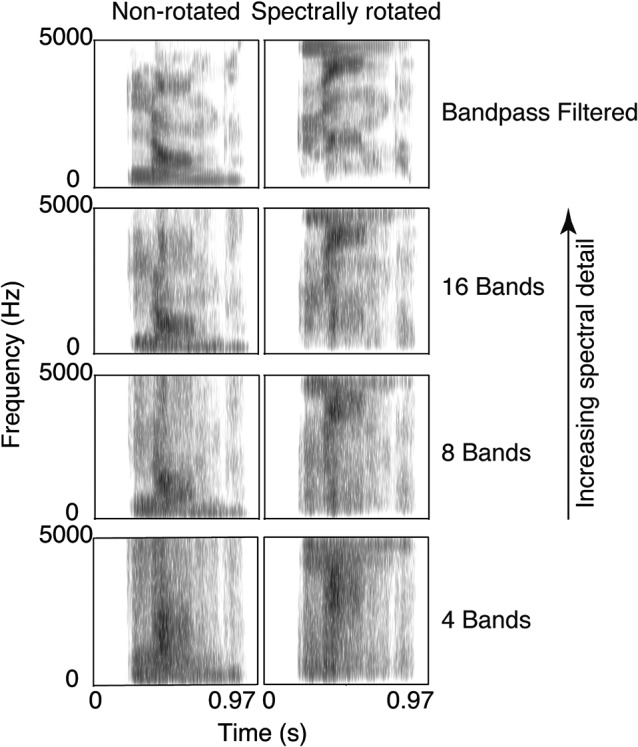
**Spectrograms of the French word “langue” (meaning tongue, or language) for the four levels of spectral detail, for spectrally rotated and non-rotated stimuli**. Stimuli illustrated with a preceding 200 ms silence.

Bandpass-filtered and NV words were also spectrally rotated, in order to create a control condition preserving the same amount of spectral information but rendering the input incomprehensible. Spectral rotation was achieved based on a procedure defined by Blesser (1972) and implemented following the procedure described by Scott et al. (2000). The speech signal was rotated around the midpoint of the frequency range of the bandpass filtered input (i.e., 2.5 kHz) and then pre-emphasized using a finite response (FIR) filter with the same long-term spectral distribution as the original input. Finally, signal was modulated by a sinusoid at 5 kHz. NV1 words were not spectrally rotated, since their spectral profile is that of white noise, i.e., flat, and would not be altered by rotation. Example NV and spectrally-rotated NV stimuli are illustrated in Figure 1.

These manipulations yielded four levels of spectral detail, crossed with the factor spectral rotation: band-pass filtered words (BP), NV16 words, 8-band noise-vocoded words (NV8) and 4-band noise-vocoded words (NV4) and their spectrally rotated homologues (rBP, rNV16, rNV8, rNV4), as well as a 1-band NV (NV1, equivalent to signal-correlated noise) control condition.

The experiment was divided into three blocks (see Section Procedure, below), for which stimulus selection proceeded as follows. Words were randomly selected from the pool of 108 non-target stimuli of each stimulus group, and allocated without replacement to one of the four non-rotated conditions (NV4, NV8, NV16, BP). Each selected word was also allocated to the spectrally rotated condition at the same level of spectral detail. Twenty seven words were allocated to each level of degradation per block. Due to the limited number of target stimuli (the 36 animal names) these were randomly allocated without replacement to each of the four levels of degradation, with a new randomization applied for each of the three blocks, thus each target stimulus appeared three times in a non-rotated condition and three times in a rotated condition over the course of the experiment. For each block, 27 words were randomly selected without replacement from the whole pool of stimuli and allocated to the NV1 condition. There were therefore 81 non-target stimuli per condition and 27 target stimuli in each condition barring NV1, for a total of 279 trials in each of the three blocks.

### Procedure

Participants were seated in front of a computer screen, at a distance of approximately 1 m, in a sound-insulated Faraday cage. The overhead light in the booth was turned off while EEG was recorded. Stimuli were delivered through ER4 noise-isolating in-ear headphones (Etymotic Research Inc., Elk Grove Village, Illinois) at a comfortable sound level, adjusted at the beginning of the experiment.

The experiment was divided into three blocks of 12.5 min and 279 trials each. Participants were instructed to listen carefully to the words and respond each time they heard an animal name by pressing a button on a response-box placed on a table in front of them with their right index finger. Brain electrical activity was recorded during the whole period of each block. Short breaks took place after the end of the first two blocks in order to check, and if necessary reduce, the impedances of the electrodes.

**Figure 2:**
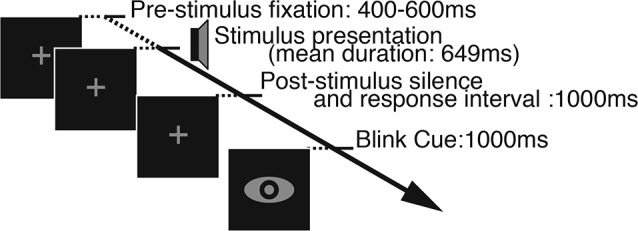
**Illustration of a single trial**. In the beginning of each trial a fixation cross appeared on the screen for a variable duration of 400–600 ms and remained on the screen as the stimulus was presented. One second of silence was inserted after the stimulus offset, during which participants could make their response, and it was followed by the blink cue whose duration was also fixed at 1 s.

Each trial began with the appearance of a fixation cross at the center of the screen. The stimulus was presented after a silent interval whose duration was randomly jittered on a uniform distribution between 400 and 600 ms after the onset of the fixation cross. The fixation cross remained on screen during the presentation of the word so as to encourage fixation and minimize eye movements. 1000 ms after the offset of the stimulus a symbol appeared on-screen for 1000 ms, indicating to participants that they could blink if they wished. By cueing blinks we aimed to ensure that any blinking-related electrical artefacts would not contaminate the recording of EEG responses to the stimuli. The structure of a single trial is illustrated in Figure 2.

### EEG recording

Brain electrical responses were recorded with a 256-electrode Electrical Geodesics HyrdoCel system (Electrical Geodesics Inc., Eugene, Oregon). Signal was recorded continuously and digitized at a sampling rate of 1000 Hz. By default, the recording system sampled at 20 kHz before applying an analog hardware antialiasing filter with a cut-off frequency of 4 kHz and downsampling the signal to 1000 Hz, and applying a software low-pass Butterworth filter with a cutoff of 400 Hz. The reference electrode was the Cz, situated at the vertex. Electrode impedances were checked at the beginning of the session and after the first and second recording blocks and were under 30 kΩ at the beginning of each block.

### Data analysis

#### Behavioral data

We computed and analyzed accuracy scores and response times for correct trials. We also analyzed the number of false alarms per condition.

#### EEG data preprocessing and artifact removal

EEG data were initially analyzed using custom Matlab (The Mathworks, Natick, MA) scripts and the freely available EEGLAB toolbox (Delorme and Makeig, 2004). Data were re-referenced to average reference and downsampled to 200 Hz (after low-pass filtering to avoid aliasing). For further analyses a set of 204 channels was analyzed (channels covering the cheeks were excluded). The three blocks per subject were concatenated and an Independent Components Analysis (ICA) was computed on the whole dataset using the Infomax routine from the Matlab-based EEGLAB toolbox (Delorme and Makeig, 2004) in order to remove blink artefacts and 50 Hz line-noise. The resulting data were filtered between 0.75 and 40 Hz using a fifth order Butterworth bandpass filter and separated into epochs starting 800 ms before the sound onset and finishing 1000 ms after. A baseline correction was applied by subtracting from each epoch the mean signal computed over 200 ms preceding the onset of the stimulus. The filtered and epoched data were scanned to detect epochs in which the amplitude between 300 ms pre-stimulus onset and the end of the epoch was higher than 75 μV or lower than −75 μV. These epochs were considered outliers and were excluded from further analysis.

Furthermore, only trials with non-target words and no button presses (i.e., false alarms) were kept for further EEG analyses.

#### Analysis of evoked activity—ERP analysis

Single-trial evoked responses were averaged for each condition separately to examine the component of neural activity being phase-locked to the stimulation event, i.e., to obtain condition-specific event-related potentials (ERPs). Data were analyzed in a time window from −100 to 1000 ms about stimulus onset.

#### Analysis of induced activity—time-frequency analysis

Using the EEGLAB toolbox, we chose a hanning-tapered short-term fast-fourier-transformation (stFFT) to compute time-frequency representations of our data in the range of the alpha band (8–13 Hz). These time-frequency power fluctuations (referred to as *TF*_α_, in μ*V*^2^) were then converted to *dB* logarithmic scale using the following equation:

(1) $$dB_{\alpha }=10\times \log_{10}\left(TF_{\alpha }\right)$$

and finally averaged across the defined frequency band. As for the ERPs, data were analyzed in a time window from −100 to 1000 ms.

#### ANOVA

The ERPs as well as the stFFT data were submitted to a repeated-measures ANOVA, including two factors: spectral detail (four levels, 4-bands, 8-bands, 16-bands and band-pass filtered), and spectral rotation (two levels, non-rotated and rotated), and including subject as a random factor. We were specifically interested in the interaction of spectral detail and spectral rotation to identify correlates of degraded word processing, which are not confounded with the factor of spectral detail. ANOVA was performed for each time point and electrode.

#### Inverse solution modeling

For the ERPs as well as the stFFT results we used the weighted minimum norm (WMN) approach (Lin et al., 2006), which belongs to the family of distributed inverse solution methods. We computed inverse solutions by applying the WMN inverse matrix to the data (5018 solution points, no regularization). The lead field model we employed was a LSMAC model (locally spherical model with anatomical constraints, for details about this approach see Brunet et al., 2011) which then in turn is applied to the MNI brain template (ICBM152) coregistered to EEG electrodes. Standard spatial electrode positions were used in all subjects, co-registered to the template MRI by adjusting the position of the nasion, inion, Cz and pre-auricular landmarks.

For the ERP data, condition and subject specific averages were inverted using the method described above and then subject to the same ANOVA procedure as the surface EEG data. To compute the inverse equivalent of the stFFT surface data in the alpha-band, we filtered the single EEG trials for each condition and subject in the alpha frequency band, inverted them and then averaged the normed (i.e., scalar) values of all single trials (similar to the approach used by de Pasquale et al., 2013). This results in a temporally resolved representation of alpha-band power distributed across 5018 solution points in the inverse space. Afterwards, these WMN inverted averages were subject to the ANOVA procedure as stated above. For visualization, results were projected onto the co-registered MNI brain template using the Cartool toolbox (Brunet et al., 2011).

## Results

### Behavioral data

**Figure 3:**
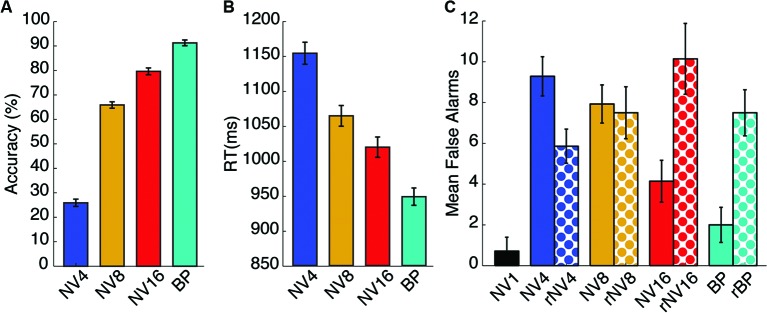
**Behavioral results**. Panel **(A)** shows the mean accuracy scores for the animal name detection task in each of the non-rotated potentially-comprehensible conditions. Panel **(B)** shows the mean response time for correct responses in the same conditions. Panel **(C)** displays the mean number of false alarms for all conditions. Error bars represent standard error of the mean corrected to be appropriate for repeated-measures comparisons, as described in Loftus and Masson (1994).

Mean accuracy values for the non-rotated potentially intelligible conditions were 26, 66, 80 and 91% for the 4-bands, 8-bands, 16-bands and bandpass-filtered stimuli respectively. Accuracy values entered in a one-way ANOVA with spectral detail as the within-subject factor (four levels: 4-bands, 8-bands, 16-bands and bandpass-filtered), which revealed a significant main effect of spectral detail (*F*_(3,39)_ = 339.931, *p* < 10^-6^, partial-**η**^2^ = 0.96). The mean accuracy value per condition is displayed in Figure 3A.

Response times for the intelligible correct trials were also computed and entered in a one-way repeated-measures ANOVA with spectral detail as the within-subjects factor (four levels, as described above). There was a significant main effect of spectral detail (*F*_(3,39)_ = 26.531, *p* < 0.001, partial-*η*^2^ = 0.67) with response latencies being faster for intelligible trials and slower for more degraded words. Response time results are displayed in Figure 3B. We did not compute accuracy values or response times for the rotated trials, as no response could be categorized as “correct” in the face of incomprehensible stimuli. Those instances where participants did respond must be considered false alarms.

We also analyzed the number of false alarms as we expected more false alarms in the unintelligible or potentially intelligible but difficult conditions. The 1-band NV words were not included in this analysis. Number of false alarms was entered a repeated-measures ANOVA with spectral detail (four-levels, as described above) and spectral rotation (two levels: spectrally rotated and non-rotated) as within-subjects factors. There was a significant main effect of spectral complexity (*F*_(3,19)_ = 5.037, *p* = 0.021, partial-*η*^2^ = 0.279) and a significant spectral complexity by rotation interaction (*F*_(3,19)_ = 8.534, *p* = 0.001, partial-*η*^2^ = 0.396). The mean false alarms per condition is displayed in Figure 3C.

### Evoked activity—ERP analysis

Average ERPs were entered into a two-way, repeated measures analysis of variance with number of frequency bands (i.e., spectral detail) and rotation as within-subjects factors. ANOVA was performed for each channel and sample point. To correct for multiple comparisons, we employed the approach of controlling the false discovery rate, FDR (Benjamini and Yekutieli, 2001) using a threshold of *p*_(FDR)_ < 0.05. In order to ensure we report robust results, we additionally disregard results involving fewer than two electrodes or having a duration of less than three consecutive sample points (15 ms). After FDR correction, we found the following main effects, at *p*_(FDR)_ < 0.05, (uncorrected *p*-values are provided here): There was a significant main effect of rotation, temporally centered around 305 ms, involving up to eight supra-threshold electrodes. At the centroparietal peak electrode 132, *F*_(1,13)_ = 57.59, *p* < 3.97*10^-6^, the effect ranged from 295 to 325 ms. A main effect of spectral detail was found in a set of seven left superior-parietal and right temporal electrodes, in a time-window ranging from 185 to 215 ms post stimulus-onset. At the centroparietal peak electrode 81, *F*_(3,39)_ = 16.5, *p* < 4.45*10^-7^.

**Figure 4:**
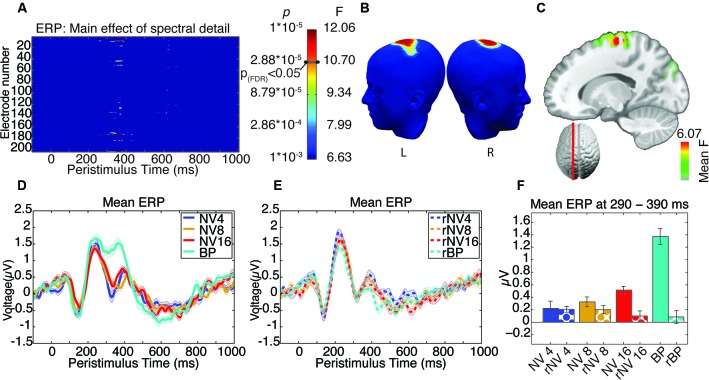
**Results of ANOVA of evoked activity**. **(A)** Electrode-by-time plot of the interaction of rotation × spectral detail thresholded at *p* < 0.001, revealing the time-window of interest between 305–390 ms. The color bar indicates the corresponding *F*- and *p*-values, the threshold for *p*_(FDR)_ < 0.05 is indicated. **(B)** Shows the topography of this effect using the same color scale as in **(A)** at the peak of the effect (345 ms). **(C)** Illustrates the localization of this effect in inverse space, displaying the mean *F*-statistic averaged over the time-window of interest. **(D)** Average time-course of this effect in a cluster of five contributing electrodes across NV conditions. **(E)** Corresponding time-courses for the rotated conditions, where the effect of spectral detail is absent. **(F)** Mean evoked activity for each condition in the significant time-window, error bars represent standard error of the mean corrected to be appropriate for repeated-measures comparisons, as described in Loftus and Masson (1994).

The focus of our analysis was the interaction between the number of frequency bands and spectral rotation which was intended to specifically reveal the EEG signatures of listening to degraded words, while controlling for the impact of spectral complexity. We observed a significant spectral detail by rotation interaction located over a set of spatially coherent central parieto-frontal electrodes, in one distinct post-stimulus time-window, from 305–390 ms, peaking at 345 ms (comprising a cluster of up to five supra-threshold electrodes around electrode 186, with maximum *F*_(3,39)_ = 21.96, *p* < 1.72*10^-8^, Figures 4A, B). It appears that the effect in this time-window is driven specifically by a significantly greater positivity in response to the bandpass-filtered words in comparison to all other categories of stimuli (see Figure 4D). In order to confirm this hypothesis, a post-hoc analysis was carried out, comparing the clear condition with all others, pooled, in a univariate ANOVA, including subject as a random factor. This showed that the clear condition was indeed significantly different from all the others *F*_ (1,13)_ = 45.303, *p* < 0.001, partial-*η*^2^ = 0.777.

### Induced activity—time-frequency analysis

**Figure 5:**
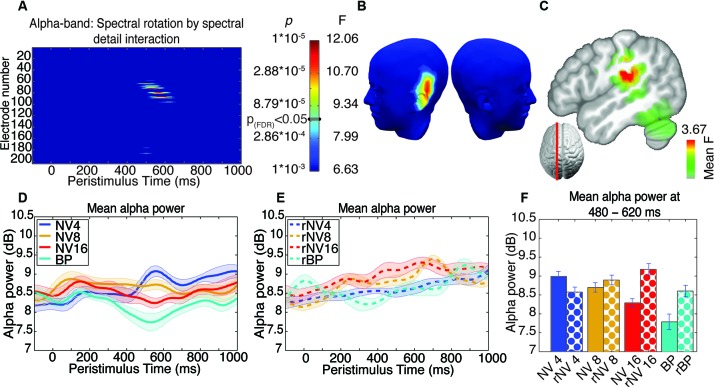
**Results of ANOVA of induced activity in the alpha-band**. **(A)** Electrode-by-time plot of the *p*-values for the interaction of rotation × spectral detail with corresponding *F*- and *p*-values, thresholded at *p* = 0.001, revealing the time-window of interest (462–633 ms). The color bar indicates the corresponding *F*- and *p*-values, the threshold for *p*_(FDR)_ < 0.05 is indicated. **(B)** Topography of this effect, using the same color scale as in **(A)** at the peak of the effect (533 ms), indicating a contribution of left-temporal sources. **(C)** Localization of this effect in the inverse space, the main source being in the left supramarginal gyrus extending into left inferior parietal and superior temporal structures, showing the average *F*-statistic over the time-window of interest. **(D)** Average time-course of this effect in a cluster of five contributing electrodes across NV conditions, demonstrating enhanced alpha-band suppression for more intelligible conditions. **(E)** Corresponding time-courses for the spectrally rotated conditions, where the effect of spectral detail is absent. **(F)** Alpha-band activity for each condition in the significant time-window, error bars represent standard error of the mean corrected to be appropriate for repeated-measures comparisons, as described in Loftus and Masson (1994).

ANOVA and correction for multiple comparisons was performed as for the ERP analysis reported above. After FDR correction, we found no significant main effects. We found a significant interaction in the alpha-band, at electrodes situated over the left temporal lobe, from 462–633 ms, with the peak of this effect found at 533 ms (comprising a spatially coherent cluster of eight supra-threshold electrodes around left-temporal electrode 75 with maximum *F*_(3,39)_ = 12.12, *p* < 9.23*10^-6^ see Figures 5A, B). This spatiotemporally coherent effect was manifest as a decrease in alpha-band power (i.e., alpha suppression) as a function of stimulus clarity for the NV but not rNV conditions (see Figures 5D, E). In contrast to the earlier effect revealed in the ERP analysis, this effect showed a graded response, suggesting sensitivity to the difficulty of comprehending words at different levels of degradation. Sidak-corrected post-hoc pairwise comparisons comparing the alpha-band power averaged over the 5 electrodes showing the greatest interaction effect in the period from 462–633 ms for the four potentially-comprehensible conditions partly support this: NV4 > NV16 (*p* = 0.021), NV4 > BP (*p* = 0.011), NV8 shows marginally less alpha power than BP (*p* = 0.063), other comparisons not significant. Although not all pairwise differences between the levels are significant, this is suggestive of increasing alpha-suppression with increasing intelligibility.

### Inverse solutions

For visualization of results in the inverse space, we performed identical statistics as those on the surface data, confined to the two temporal windows showing FDR-corrected effects in both the ERP and the induced activity. The results are presented in Figures 4C, 5C showing the resulting *F*-values averaged over the time-window of interest.

For effects on evoked activity, the source of the corresponding effect (i.e., the interaction between spectral complexity and rotation) in the time window from 300–400 ms with a peak at approximately −13, −27, 63 (MNI co-ordinates) principally located in medial precentral gyrus (BA6, extending posteriorly into BA4). This effect is shown in Figure 4C.

The source corresponding to the interaction between spectral complexity and rotation in the alpha band (time window 450–650 ms) was localized to the left superior temporal lobe/inferior parietal lobe. The maximum effect was located at −46, −27, 20 (MNI co-ordinates), and spanned the posterior reaches of the left supramarginal gyrus, and covered the superior posterior temporal gyrus. The effect is presented in Figure 5C.

## Discussion

Behavioral data demonstrate that the rNV conditions were unintelligible to the participants in this study, while the intelligibility of the NV conditions increased as a function of the number of frequency bands used, as previously demonstrated (e.g., Shannon et al., 1995; Davis and Johnsrude, 2003). Although we did not obtain responses for every trial, we are confident that the d’ measures established for the target-detection task reflect the mean level of intelligibility of each of the conditions employed.

Our analysis of the EEG data revealed two time-periods in which the brain response is significantly affected by the potential intelligibility of a stimulus. In the earlier one of these, we show an effect in evoked activity that appears to relate to the early identification of clear stimuli as a special category. Its timing and its topography suggest that we may be detecting modulation of the P300 component (for a review of the P300, see Polich, 2007). The P300 is usually apparent as a positivity in the midline parieto-frontal electrodes and is observed in tasks, such as the animal name detection task used here, that require stimulus discrimination. Its appearance has been suggested to be an index of the occurrence of stimulus events sufficiently important to inhibit concurrent processes, and has therefore been associated with attention and memory processes (Polich, 2007). In the present case, it could reflect the fact that deciding whether the stimulus is a target or not is substantially easier when the word is more easily intelligible. Another possibility is that because the clear stimuli are categorically different from all others, this relatively early effect results from an oddball effect (Polich and Margala, 1997). This is also supported by the fact that the probability of a clear stimulus being presented was relatively low, at only 13%. The fact that this evoked effect seems to be rather central than left-lateralized could also be seen to support the view of this component being rather a general cognitive component than one specific to language processing.

We did not find effects in the evoked responses that we can more specifically ascribe to comprehension processes. This may be the result of the inherent difficulty of time-locking EEG responses to specific events during auditory word processing. Although we used the acoustic onset of the stimuli as our marker for the beginning of each epoch, given that we are interested in comprehension processes rather than auditory processes, this may not be the best point. Ideally, we might have used the recognition point determined using (for example) gating (Grosjean, 1980). This issue, of course, applies to all studies of spoken word perception that use measures with a high temporal resolution.

During a second time-window, we showed a significant interaction between spectral rotation and spectral complexity on induced activity—specifically the alpha band, reflecting increased alpha suppression as a function of increasing spectral complexity in the NV (i.e., potentially comprehensible) and not the rNV (always unintelligible) stimuli. This finding is superficially similar to that reported by Obleser and Weisz (2012), who demonstrated a connection between alpha-band suppression and intelligibility of stimuli. However, by including the spectrally rotated stimuli as an unintelligible control condition we extended this finding by confirming that the relationship between alpha-suppression and intelligibility is almost certainly the result of brain mechanisms that extract meaning from words, rather than the result of purely acoustic analysis of more vs. less complex auditory input. Furthermore, the effects we find occur earlier than those reported by Obleser and Weisz (2012). This may be the result of our having employed exclusively monosyllabic words rather than the mixture mono-, bi- and trisyllabic words they employed.

As discussed in Section Introduction, the functional role of auditory alpha is unclear, although it is increasingly becoming a focus of attention in the field of speech perception. At present, the relevant evidence seems to indicate that alpha-power suppression is related to comprehension, and it has been suggested that this could relate to the role of alpha-band activity in suppressing higher-frequency oscillations. It may be the case that alpha-band activity reveals an inhibitory process that suppresses processes whose occurrence is indicated by the presence of higher-frequency oscillations (Osipova et al., 2008). Thus, alpha-suppression may reflect the release of inhibition of processing. In the present case, where alpha-suppression reflects the intelligibility of auditory input, it is highly likely that the process indexed by this effect is related to word identification and comprehension. This effect is present in the time range of the N400 complex, which is related to semantic processing (Kutas and Hillyard, 1984).

Increased alpha-suppression for more easily comprehensible words during this time window is consistent with the idea that this effect reveals the on-average greater semantic processing in the easier compared to the harder conditions. However, a less prosaic explanation of this effect would be that the relatively higher alpha power in the more challenging conditions reflects increased functional inhibition related to the demands placed on attention and working memory by the challenge of comprehending acoustically degraded speech. Indeed, several studies have shown that the ability to comprehend degraded speech is influenced by working memory capacity (Ronnberg et al., 2008, 2010; Zekveld et al., 2009, 2011, 2012; Rudner et al., 2011) and selective attention (Shinn-Cunningham and Best, 2008; Wild et al., 2012b). It is possible that this effect is the manifestation of the inhibitory networks that are required to maintain selective attention and working memory processes to support the comprehension of degraded speech. A possibility raised by Obleser and Weisz (2012) is that the increase in alpha-suppression observed in response to less degraded stimuli is not necessarily increased suppression as a function of increased intelligibility, but increasing alpha-power as stimulus intelligibility falls. If this is the case, then the effect may be explained if alpha is considered to be a gating mechanism that inhibits other cognitive processes locally while challenging speech comprehension takes place.

Our data indicate that—in comparison to baseline—there is indeed an increase in alpha-power in response to the most challenging NV condition, indeed suggesting that the role of temporal alpha may be to actively regulate the engagement of downstream cognitive processes (confirmed by one-tailed posthoc *t*-test, *p* = 0.017). In the present case, it is possible that suppressing the final stages of word-identification processes (i.e., lexical access or semantic access) is a way of improving word-identification accuracy by enabling additional time for processing of the acoustic input. The possibility that the identification processes are delayed as a function of degradation can be investigated in future studies.

The effect at this time was localized to a region incorporating the left supramarginal gyrus (SMG), left inferior parietal lobule, and the posterior left superior temporal gyrus. This approximate localization situates the effect in a region that has been considered by some authors to be part of Wernicke’s region (Geschwind, 1972; Bogen and Bogen, 1976) and has also been associated with sensorimotor integration during speech comprehension (Hickok and Poeppel, 2007). A role for sensorimotor integration during degraded speech comprehension would be consistent with the notion that, when confronted with degraded speech, the speech comprehension system falls back on higher-level (top-down) mechanisms to help resolve the degraded acoustic input. In this case, since very little linguistic information is present in the degraded monosyllabic stimuli, such information could potentially be articulatory in nature (e.g., as suggested by Hervais-Adelman et al., 2012). Nevertheless, beyond implicating this region in degraded speech processing, evidence for its exact role is scant.

The localization of the source of this alpha-suppression effect using a weighted minimum norm estimate was rather different to that shown by Obleser and Weisz (2012) using a beamformer approach. While we show relatively localized sources, they showed more distributed sources incorporating anterior temporal regions, parietal lobes, right dorsolateral prefrontal cortex and left inferior frontal regions.

Another potentially fruitful line of enquiry concerns the link between alpha-suppression and the pulvinar nucleus of the thalamus. Although we do not directly observe any effects in the pulvinar in this study, an investigation by Erb et al. (2012) implicated this structure as being linked to individuals’ ability to learn to comprehend NV speech. Activity in the pulvinar nucleus has been suggested as a driver of alpha suppression (Lopes da Silva et al., 1980; Liu et al., 2012), and is also linked to attentional control in vision (e.g., Kastner et al., 2004; Smith et al., 2009) and audition (Wester et al., 2001), while attention has been found to affect the cerebral processing of NV speech (Wild et al., 2012b). Although at this stage this potential link is highly speculative, we would suggest that the thalamo-cortical networks implicated in alpha-band activity are worthwhile targets for investigations of speech comprehension.

Interestingly, we do not find regions in which alpha-suppression shows the opposite response to intelligibility (i.e., alpha is more suppressed when stimuli are harder to understand). This is inconsistent with the fMRI literature, in which many studies have demonstrated the recruitment of additional brain areas when speech perception is challenging due to acoustic degradation compared to when it is clear. For example, several studies have demonstrated that left inferior frontal gyrus regions are engaged when acoustically degraded speech is presented (Davis and Johnsrude, 2003; Giraud et al., 2004; Obleser et al., 2007; Hervais-Adelman et al., 2012; Wild et al., 2012b), others have shown increased recruitment of anterior and posterior temporal lobe structures in the face of acoustic degradation (Scott et al., 2000; Narain et al., 2003; Evans et al., 2013). We would have expected to see such effects reflected in the EEG signature of degraded speech processing. That we do not find effects in this direction in these data is puzzling and demands further investigation. However, generation of alpha oscillations can differ considerably from site to site, even within the visual system. This has been reported for example with regard to originating layers of alpha and even relationship of the measured alpha oscillations to behavioral performance (Bollimunta et al., 2008).

Another interesting detail is that the sites of our observed major effect in the alpha-band and the sites of highest spectral power in the alpha-band do not spatially coincide. As is commonly known, occipital electrodes exhibit much higher resting-state alpha power than the remaining electrodes. The left-temporal cluster identified in our study shows only moderate prestimulus alpha-amplitude. Yet its modulation is highly significant. This demonstrates that while such alpha modulation might be more difficult to identify, it can nonetheless be extracted.

In general, the link between alpha rhythm and language/word processing is relatively sparsely described. While there seems to be some consensus about auditory alpha/tau (see Lehtela et al., 1997), the idea of higher-level language-related alpha oscillations is still quite recent (e.g., Obleser and Weisz, 2012) and highly exciting. Whether this points to an even more universal role of alpha oscillations than previously envisioned remains to be seen and will require further investigations in more diverse experimental settings.

## Conclusion

Taken together, the data presented above suggest that the auditory system can rapidly differentiate easily-comprehensible speech (approximately 300 ms after stimulus onset), and that potentially-comprehensible degraded speech can begin to be processed differently to incomprehensible speech as early as 480 ms after stimulus onset. We demonstrate that alpha-band power in the left temporal lobe is modulated by stimulus intelligibility. Spatial and temporal features of this effect seem to suggest this effect indexes the ease of access to word meaning. Our paradigm ensures that the alpha-band desynchronization we observe is not simply related to increasing spectral detail in the stimuli, and thus confirms that alpha-band desynchronization at left temporal sites reflects word intelligibility. These findings provide insights into the neural mechanisms of degraded speech perception, and may be taken to suggest that one strategy in compensating for acoustically-degraded input may be to suppress recognition processes that ordinarily take place rapidly and automatically, in order to permit additional processing to be carried out on the degraded signal.

## Conflict of interest statement

The authors declare that the research was conducted in the absence of any commercial or financial relationships that could be construed as a potential conflict of interest.

## Footnotes

^1^http://www.lexique.org, New et al. (2001)

^2^www.mrccbu.cam.ac.uk/~maarten/match.htm, Van Casteren and Davis (2007)

^3^http://audacity.sourceforge.net/
