# Corrigendum: Left temporal alpha-band activity reflects single word intelligibility

**DOI:** 10.3389/fnsys.2014.00047

**Published:** 2014-04-01

**Authors:** Robert Becker, Maria Pefkou, Christoph M. Michel, Alexis G. Hervais-Adelman

**Affiliations:** ^1^Functional Brain Mapping Lab, Department of Fundamental Neuroscience, University of Geneva, University Medical School Geneva, Switzerland; ^2^Brain and Languge Lab, Department of Clinical Neuroscience, University of Geneva, University Medical School Geneva, Switzerland

**Keywords:** speech intelligibility, degraded speech, noise-vocoding, alpha oscillations, left inferior temporal cortex

Figure 5 of the article by Becker et al. (2013) contained a minor error, which we hereby rectify. In the original figure at the bottom left of panel C the indication of the sagittal section used for display of the inverse solution is incorrect. We therefore re-submit Figure 5 with the correct cross-section for subpanel C.

**Figure 5:**
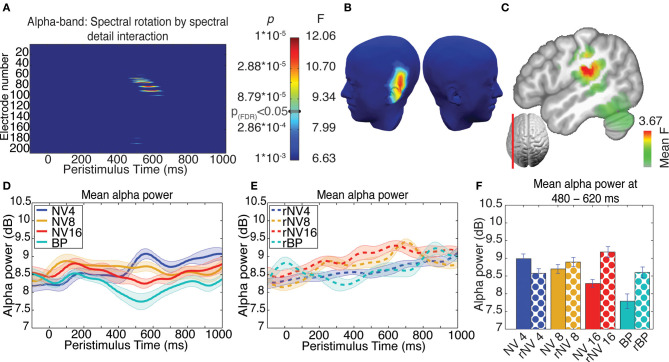
**Results of ANOVA of induced activity in the alpha-band. (A)** Electrode-by-time plot of the *p*-values for the interaction of rotation × spectral detail with corresponding *F*- and *p*-values, thresholded at *p* = 0.001, revealing the time-window of interest (462–633 ms). The color bar indicates the corresponding *F*- and *p*-values, the threshold for *p*(FDR) < 0.05 is indicated. **(B)** Topography of this effect, using the same color scale as in **(A)** at the peak of the effect (533 ms), indicating a contribution of left-temporal sources. **(C)** Localization of this effect in the inverse space, the main source being in the left supramarginal gyrus extending into left inferior parietal and superior temporal structures, showing the average F-statistic over the time-window of interest. **(D)** Average time-course of this effect in a cluster of five contributing electrodes across NV conditions, demonstrating enhanced alpha-band suppression for more intelligible conditions. **(E)** Corresponding time-courses for the spectrally rotated conditions, where the effect of spectral detail is absent. **(F)** Alpha-band activity for each condition in the significant time-window, error bars represent standard error of the mean corrected to be appropriate for repeated-measures comparisons, as described in Loftus and Masson (1994).
